# Location, location, location: utilizing pipelines and services to more effectively georeference the world's biodiversity data

**DOI:** 10.1186/1471-2105-10-S14-S3

**Published:** 2009-11-10

**Authors:** Andrew W Hill, Robert Guralnick, Paul Flemons, Reed Beaman, John Wieczorek, Ajay Ranipeta, Vishwas Chavan, David Remsen

**Affiliations:** 1 University of Colorado Museum of Natural History and Department of Ecology and Evolutionary Biology, University of Colorado Boulder, Boulder CO 80309-0265, USA; 2 Australian Museum, 6 College St Sydney 2010, New South Wales, Australia; 3 Florida Museum of Natural History, University of Florida, Gainesville FL 32611, USA; 4 Museum of Vertebrate Zoology, University of California, Berkeley CA 94720, USA; 5 Global Biodiversity Information Facility Secretariat, Universitetsparken 15, DK-2100, Copenhagen, Denmark

## Abstract

**Background:**

Increasing the quantity and quality of data is a key goal of biodiversity informatics, leading to increased fitness for use in scientific research and beyond. This goal is impeded by a legacy of geographic locality descriptions associated with biodiversity records that are often heterogeneous and not in a map-ready format. The biodiversity informatics community has developed best practices and tools that provide the means to do retrospective georeferencing (e.g., the BioGeomancer toolkit), a process that converts heterogeneous descriptions into geographic coordinates and a measurement of spatial uncertainty. Even with these methods and tools, data publishers are faced with the immensely time-consuming task of vetting georeferenced localities. Furthermore, it is likely that overlap in georeferencing effort is occurring across data publishers. Solutions are needed that help publishers more effectively georeference their records, verify their quality, and eliminate the duplication of effort across publishers.

**Results:**

We have developed a tool called BioGeoBIF, which incorporates the high throughput and standardized georeferencing methods of BioGeomancer into a beginning-to-end workflow. Custodians who publish their data to the Global Biodiversity Information Facility (GBIF) can use this system to improve the quantity and quality of their georeferences. BioGeoBIF harvests records directly from the publishers' access points, georeferences the records using the BioGeomancer web-service, and makes results available to data managers for inclusion at the source. Using a web-based, password-protected, group management system for each data publisher, we leave data ownership, management, and vetting responsibilities with the managers and collaborators of each data set. We also minimize the georeferencing task, by combining and storing unique textual localities from all registered data access points, and dynamically linking that information to the password protected record information for each publisher.

**Conclusion:**

We have developed one of the first examples of services that can help create higher quality data for publishers mediated through the Global Biodiversity Information Facility and its data portal. This service is one step towards solving many problems of data quality in the growing field of biodiversity informatics. We envision future improvements to our service that include faster results returns and inclusion of more georeferencing engines.

## Background

A major goal for biodiversity informatics is to provide high quality data for biodiversity and related life science research, including phylogenetics, genomics, ecology, as well as a broad array of downstream use, including earth sciences (e.g, climate change research), resource management, and education. One of the most fundamental aspects of any biodiversity occurrence record is a locality description, whether that description is textual or in a more readily analytical format like latitude and longitude [1,2]. For most legacy records, locality descriptions are textual and therefore an interpretive process is needed to assign geospatial coordinates. This process is referred to as retrospective georeferencing [3]. Performing retrospective georeferencing is a core task in biodiversity informatics because it increases the quality and usability of a specimen occurrence record, providing a means to link those records to geographical and environmental data in a geographic information systems context.

A retrospective georeference is essentially a hypothesis that describes (using quantitative map coordinates) an interpretation of a locality description based on best available geographic information. The process of automated georeferencing should be repeatable, and should always return the same georeference (latitude, longitude, reference system, coordinate uncertainty) for a given textual string, interpretation algorithm, and base geographic data. As methods of interpretation evolve and geographic data improve, a georeference is subject to revision, just like any other hypothesis. Retrospective georeferencing as part of an automated workflow simplifies and improves the efficiency with which versions of georeferences can be managed and utilized.

The retrospective georeferencing process is an immense undertaking given that only a fraction of the legacy records from natural history repositories already have geospatial coordinates. Even worse, because best practices for georeferencing have only recently been developed [4], existing retrospective georeferences are often poorly documented with respect to precision, accuracy, uncertainty, reference system, sources, methods, and verification status [5]. Without effective georeferencing it is difficult to determine a given occurrence's fitness for use to answer a given question.

Multiple generic georeferencing services exist that provide digital results (Metacarta, Yahoo! Geoplanet) and a few are specifically motivated by the retrospective georeferencing of biodiversity data (GEOLocate, ; BioGeomancer, ). Of these, the BioGeomancer service allows users with minimal knowledge of the georeferencing process to quickly generate standardized georeferences of high quality and their associated uncertainty. Users need not start from scratch, developing a methodology that may not reflect community developed best practice. Furthermore, users can take advantage of a standardized process - where the same results will be returned for duplicate records across all biodiversity collections - and a highly developed georeferencing algorithm targeted at biodiversity data [5].

While large-scale collaborative georeferencing projects, using best practices, have been accomplished for certain taxonomic groups such as mammals (MaNIS, ), birds (ORNIS, ), and reptiles and amphibians (HerpNet, ), biodiversity records, in general, are not as likely to be associated with high quality georeferences. Even with automated or semi-automated workbenches, the task of georeferencing over one billion biodiversity records in a reasonable amount of time is immense and daunting. In the past, the MaNIS project achieved 16.6 (± 8.3) georeferences per hour [6]. Now, the BioGeomancer project doubles this rate [7]. In order to fully utilize the potential of the georeferencing advancements, we need further improvements in the speed of submitting georeferencing jobs and reporting results.

A partial solution to the challenge of large-scale georeferencing will be to not only provide automation for certain steps of the process (e.g., interpretation of textual localities to geographic coordinates), but also to create pipelines integrated with human interaction where those tools are constantly operating on the growing set of biodiversity occurrence records as they become available [8]. These pipelines can be developed to automatically feed digital occurrence data from publishers to the BioGeomancer service for georeferencing. The results of the process are stored for data publishers to review and integrate with their original records.

Here we discuss a prototype system to provide data contributors, and ultimately any user, with standardized and high quality georeferenced information for occurrence records that:

• have not been georeferenced previously

• have been georeferenced but lack record of georeferencing method or process

• have been georeferenced but lack uncertainty calculations

• contain one of several common errors (e.g., transposed latitude and longitude)

• were initially georeferenced without taking into account best practices

Once in place, the system will offer a value-added service to many publishers who may not have the capacity to georeference records themselves.

Building such a georeferencing pipeline as a toolkit required solving a series of technical challenges. In the rest of this paper we describe and discuss possible solutions to these challenges and present the particular implementations we employed while developing our application. In particular, we discuss the following challenges: 1) developing an opt-in system for data publishers; 2) developing systems to index GBIF and publisher data for purposes of data improvement; 3) developing effective and user-friendly systems for returning the georeferencing outputs to data publishers so they can easily check progress and manage the resulting new or improved georeferences.

## Results

### Introduction to workflow

The system we discuss in this paper was designed with simplicity in mind. After a data publisher registers the access point to their biodiversity collection, their records are harvested and sent to the BioGeomancer web service. BioGeomancer services automatically assign an uncertainty radius around latitude-longitude coordinates based on the best practice documented in Chapman and Wieczorek (2006). The point-radius method is an appropriate initial focus because it is encoded into the Darwin Core standard  and it utilizes only fields that most publishers can currently accommodate. A more complex geospatial engine is not required. The results are returned and stored in a database and data publishers are notified that the new georeferences are available. Data publishers may access the georeferenced records and associated metadata (harvest information, georeference methodology, etc.) via a secure website. In a future release, a secure service will expose the data as individual records or record sets based on quality of georeference, date of harvest, or common error types identified for programmatic access. Using these filters, data publishers can evaluate records and choose whether to incorporate the computed coordinates back into their databases directly or not.

A downstream goal is to work with data publishers to help them update their databases. By working with them to integrate georeferencing results back into their main databases, we can also increase georeferencing capacity within the network of data publishers. Additionally, we hope to work with data publishers to develop tools for automated record retrieval. We see this as being an important stage in the effort towards correctly documenting georeferencing changes and methods at the sources or original records.

### Developing a voluntary data publisher service

Proper maintenance of data custodianship is of primary importance for a harvesting service targeted at biodiversity collections data. To address this issue, we have designed the service to work behind a password protected, opt-in only, web-based environment. The opt-in process was initiated by working with GBIF to send an invitation letter to the data publishers based on contact information in GBIF-registered datasets. The letter informed the publishers that a georeferencing service was available and requested permission to harvest their data in order to perform automated georeferencing. We have completed a round of testing using these data. As a result, we have designed our system to be managed by the primary collections administrators through the entire process from portal registration through data retrieval. This is detailed below.

We developed the BioGeoBIF harvesting and results-return service within the Drupal web development framework (Drupal.org), an open source content management platform. The components of BioGeoBIF are deployed as a set of Drupal Modules, which allows us to quickly move, expand, or reinvent the service to fit future needs. Our prototype service can be found at . The site has three-tier management system. These tiers consist of an overall site administrator, group administrators, and users. Visitors to our site will first encounter a welcome screen including information about user and group registration. The first step for any visitor will be to determine if their digital collections access point has already been registered in the service. This information can be found in our Publisher Listing area or by simply attempting to register the georeferencing data access point. A visitor can submit a new registration request by providing their contact information as well as information regarding the data portal in an easy to use form. Registration of new collection access points will be handled by the overall site administrator. Following acceptance, login information will be automatically generated and sent to the registrant. Registrants who create a new collections access point node in Drupal become group administrators for that node and can then authenticate other registrants to become users.

Once registered, each data access point is managed within its own group; harvesting and georeferencing results are only viewable by approved members of the group. To join an existing group, members can either be invited or request invitation by group administrators and may join as many groups as they are involved with. Groups can invite as many or as few members as they need, including everyone from database administrators to undergraduate collaborators. This design removes group management responsibility from BioGeoBIF administrators and gives it to the data custodians. Group members are given control of which resources served at the access point are harvested, and are able to quickly sort and download results.

Structuring the service as a set of data access points with resources managed as a set of groups by group members allows us to streamline many of the data quality improvement processes. One efficiency solution we employ is to remove duplicate locality descriptions, tying each harvested catalogue number to a unique locality key. By utilizing this approach, we are able to version both the harvesting and georeferencing architecture. These metadata will allow users to continually improve the quality of their georeferencing based on the versions of the programs (e.g., BioGeomancer version 1.2) used to generate them.

### Data processing

The first step to generating georeferences is to harvest new data from publisher access points. Currently there are still no ubiquitous harvesting tools that can handle the diverse types of biodiversity data transmission protocols (BioCASE; [9], DiGIR; [10], TAPIR; , etc.). BioGeoBIF first attempts to determine the supported protocol: DiGIR, TAPIR, BioCASE, or other. We utilize newly developed code, drawing on methods encoded in TapirTester , to store the type, structure and name that describe each access point. This information can be used to create resource management pages within the group, and begin harvesting records from each resource.

**Figure 1:**
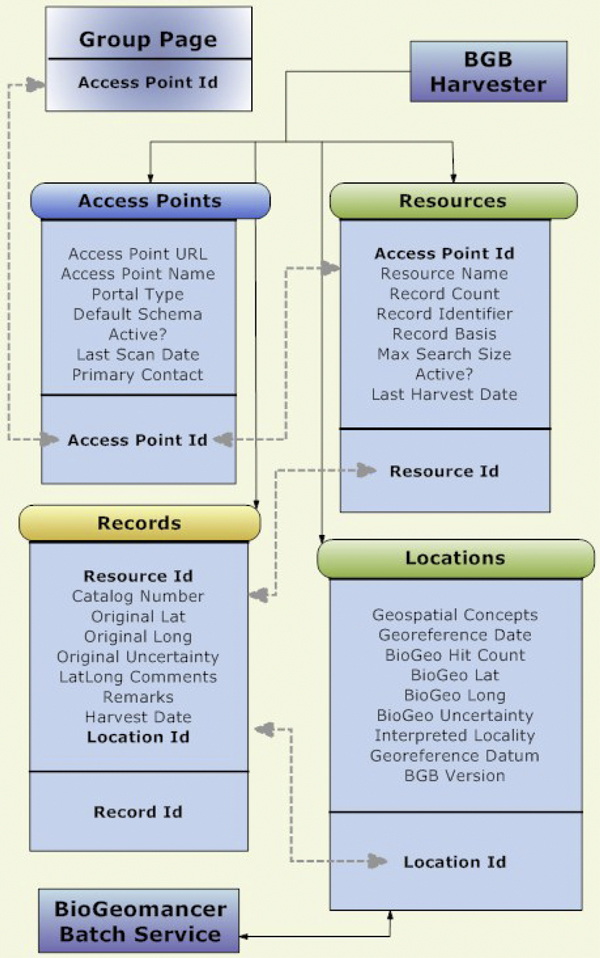
**Simplified BioGeoBIF Data Structure**. The BioGeoBIF indexer collects data from each access point and stores it in each of four tables: Access Points, Resources, Records, and Locations tables. From the relational information in these tables, the service frontend can display and retrieve harvested and georeferenced data for registered users.

We collect only a minimum amount of information associated with each resource: CollectionCode, CatalogNumber, and all DwC spatial extension fields. Most of the biologically relevant fields are not necessary or collected for the purposes of this project. We hope to extend this framework as a model to other data improvement projects, e.g., for identifying taxonomic name errors [11]. The harvested data for each record is stored in a primary database table (*Records*, Figure 1), and each unique geographic description is stored in a second table linked by a unique locality id field (*Locations*, Figure 1). Many common geographic locations will occur in biological collections from diverse sources; therefore, some updated georeferences for newly registered data portals will become available as soon as they are indexed.

BioGeoBIF will continue to send sets of geographic descriptions to the BioGeomancer web service for batch georeferencing as long as unprocessed data exist. To minimize the load on the BioGeomancer web service, we only run these requests during periods of slow traffic at BioGeomancer. The process and standards that BioGeomancer employs are given in detail in Guralnick et al. (2006), but basically, BioGeomancer interprets the text and divides it into simple "clauses", each of which can be described spatially using a combination of feature name lookups and geometric algorithms. The spatial intersection of all clauses for a given textual description usually provides one or more final georeferences. When complete, the results for the set of records are stored in the database discussed above. Any groups affected by the update are notified through the Drupal site (and in the future through email or content publication notification methods like Really Simple Syndication (RSS) subscriptions), that new results are available for a particular resource. These results only currently represent the latitude, longitude, and uncertainty that we can generate from existing best practice available. As such, it is important that we store metadata about versions of the BioGeoBIF and BioGeomancer services used to obtain each georeference. A near term goal of BioGeoBIF is to begin to allow community verification of georeferencing results, thus adding a higher level of quality control that can be shared among all users.

We aim to provide a set of error detection tools to improve high quality georeferencing that already exists in resource databases but may have suffered errors during data entry or other data manipulations. By comparing the BioGeomancer best georeferences to existing georeferences, we can often detect two common errors: switched latitude and longitude fields or improperly negative or positive decimal degrees. If detected, we can then notify the data publisher of the specific records where each of the error types occurs.

We have also begun to give users automatically generated Structured Query Language (SQL) statements that, if used, will quickly and seamlessly update their local databases to reflect the new georeferences. The SQL statements are customizable so that group users can easily map our result format to their own SQL schema. We can also provide SQL statements for other types of action including updates that provide simple fixes to specific georeference errors (e.g., transposed latitude and longitude) discussed above. Generating SQL statements is another way to provide a service that helps data owners but leaves control over databases with those owners. One pitfall to automatic SQL statements is the risk of changing data where BioGeoBIF and BioGeomancer have not actually improved upon the original data. To avoid this, we offer the users various methods to sort results by uncertainty and ambiguity of results to increase the speed at which they can find the high quality results they approve for repatriation.

### Result retrieval

**Figure 2:**
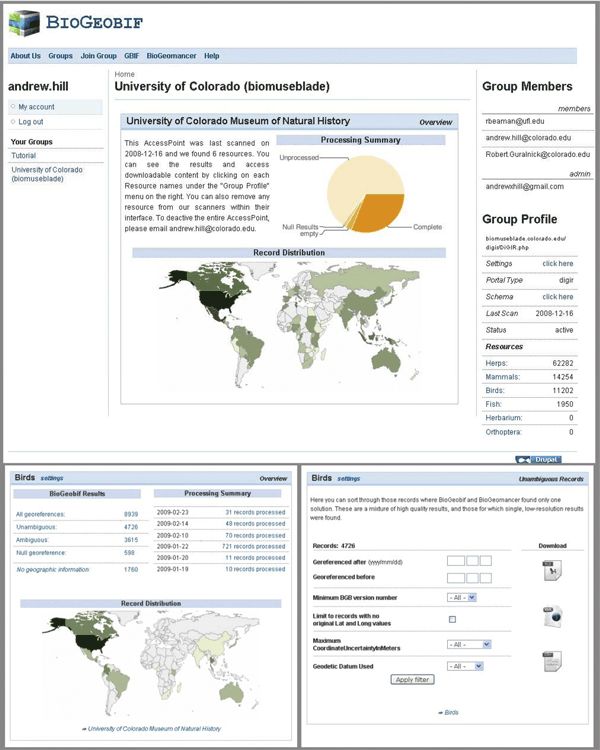
**Group Management Screen Shot**. Top: The main page displayed to an approved user when they log on to the BioGeoBIF service. In this case, the University of Colorado access point is hosting six resources. Bottom Left: After a user clicks on the *Birds *resource link, the center column changes to display information regarding just the georeferencing progress for the *Birds *resource. Bottom Right: By clicking on *Unambiguous *the user is given a set of search parameters they can use to narrow down the data they wish to download. Then they can download the results in Excel, CSV, or XML format.

Results from the BioGeoBIF service are currently made available to users through their Resource Management Page (Figure 2). Users can quickly see the proportion of records that BioGeoBIF has processed and divided into three result types: unambiguous georeferences, ambiguous georeferences, and null georeferences.

**Unambiguous georeferences **are those records where BioGeomancer found only one possible latitude and longitude for the textual description. The unambiguous records will contain a latitude, longitude, and uncertainty for users to evaluate and download for incorporation into the original data source. Not all records with unambiguous results are necessarily desirable to the data publishers. For example, in some cases errors in the gazetteer used may give unambiguous results that are incorrect.

**Ambiguous georeferences **are those records for which BioGeomancer found multiple possible georeferences. Currently, users can sort these records and download them for local evaluation. In the near future, we plan to create a more seamless upload from the BioGeoBIF management service to the BioGeomancer batch service so that publishers can utilize the mapping and other capabilities of BioGeomancer to further refine and isolate the best georeferences.

**Null georeferences **are those cases where BioGeomancer was unable to generate a georeference based on the descriptions alone. We make these results sortable and available to the users in a similar manner to the unambiguous and ambiguous records.

To sort through the BioGeoBIF results, users can enter various search terms. First they can restrict results to records georeferenced before or after a certain date. This will help users to keep from evaluating the same results multiple times. Second, they can sort by the service version number. In the future, new users may wish to only accept records from the most recent iteration of the georeferencing service. Maximum georeference uncertainty is the next field for sorting records. We see this as one of the best methods for targeting georeferences of the highest specificity. In addition to our search methods, we also make records available in a variety of formats (Excel, XML, and CSV). In each case, users can employ their own local methods of evaluating data prior to accepting and incorporating it into their local databases. A primary objective of all georeferencing projects is to create tools that data custodians will use to improve data at its source. As such, the members of the BioGeoBIF project hope to incorporate outreach and collaboration with those custodians to make this happen.

## Conclusion and future directions

BioGeoBIF provides an interactive service that allows data publishers to quickly and automatically improve geospatial quality of biodiversity data. On the whole, the georeferencing task is a daunting one faced by all biodiversity data publishers, but one that enormously increases the potential for broader use of the data. Utilizing the georeferencing framework developed through the BioGeomancer project, we have developed one of the first examples of services that help create higher quality data for publishers mediated through the network of the Global Biodiversity Information Facility and its data portal. Although the primary objective of our service is to provide the methods to ultimately be able to georeference possibly billions of records, we also seek to drive development of georeferencing technology itself. Below we touch on a number of key future directions. We first discuss issues and possible resolution regarding speed of georeferencing. Next we discuss the importance of versioning and how that can support continued refinement of georeferencing quality. We then discuss how the workflow system we have developed can be extended for georeferencing and potentially redeployed to handle other data quality issues.

A major bottleneck in semi-automated georeferencing workflows is the performance of the georeferencing engines and the computational load that the georeferencing places on the servers. Several methods could be implemented to address the speed of the BioGeomancer service directly. The BioGeomancer service could be, 1) replicated across multiple mirrors; 2) implemented in a server cloud or dedicated cluster; 3) undergo future improvements to algorithm efficiency.

A related issue is that some georeferencing engines may be more suitable for certain types of records in terms of speed and quality of georeferencing. GEOLocate, for example, has been shown to provide particularly high quality georeferencing results for records collected from water bodies and other distinct geographic feature types (river/road crossings). Ideally, records meeting a set of criteria should be sent to the appropriate service. The drawback to this method is that not all engines follow the best practices for determining uncertainty for calculated georeferences. Before we can combine these different georeferencing algorithms into applications, appropriate methods must be developed to assess results from such combined analyses. This could be mediated by proper BioGeoBIF version documentation and inclusion of this information in the *georeferencing remarks *field available to data publishers.

Retrospective georeferencing is still evolving as a process and because of that, georeferencing is not a static, one time output. Therefore, versioning is extremely important. Our versioning system will allow users to directly track the "up-to-dateness" of their georeferenced data. It will also allow them to determine what sorts of improvements can be made by reanalyzing their data. In the near future, we will allow programmatic access and sorting of a group's georeferencing results through our service. We also see a need for building more cross-community verification services. The objective would be to allow users to verify the quality of a georeference and for any other user to view, comment, and utilize that verification information. We can build much of this directly into our BioGeoBIF web interface. Furthermore, we hope to link the system with social networking tools, where users and groups across the biodiversity community can share georeference verifications and modifications. This could also directly improve the gazetteer information currently available. Bringing the biodiversity informatics into a social network environment could also enhance the speed at which we identify problem areas, develop solutions, and enhance the information stored with our primary data. Such collaborative georeferencing approaches are already being developed (e.g., GEOLocate Community Edition: ).

Versioning becomes even more important as the very nature of georeferencing representation may change in the future. In particular, the method of reporting record location and measurement of coordinate uncertainty is an area of continued research. Currently, georeferences are reported as a centroid with a radius of error. But as biodiversity informatics tools become more geospatially sophisticated, we should begin to incorporate new methods to calculate and visualize geographic uncertainty, such as probability surfaces [12,13]. Storing probability surfaces is not directly supported in any current data portal software nor can other programs easily ingest these data until probability surface implementations become more widely available. However, with probability surfaces, uncertainty is inherent in a more detailed representation of the data (i.e., accounting for geographic breaks such as water bodies in the probabilities). We feel that these representations will also lend themselves to a better understanding of the spatial arrangement of biodiversity.

High throughput georeferencing for the world's biodiversity collection data is the primary goal of our service. However, we also believe the same service could be used to georeference automatically extracted and tagged locality information from the biological primary literature. Services such as Plazi.org [14,15] are already functional and we anticipate that data from Plazi.org will be shipped to BioGeoBIF for georeferencing either prior to or after being published to GBIF. In this way, managers of tagged literature data can use our user reporting and updating services just like any other data provider. We also hope to use the frameworks for group and user management, data harvesting and storage procedures, and result returns as the basis of a wide variety of data improvement methods. Problem detection and improvement methods for taxonomic name standards, date formatting standards, and other issues that may arise can all be incorporated into our workflow. The future of biodiversity informatics is going to rely heavily on automated workflows and analyses [16] that can assemble and analyze raw data and convert them into either high quality data or into summary information products.

## Methods

Indexing code to access publisher records or those stored in the GBIF data cache, and code to access the BioGeomancer web service were written in PHP and are available through the BioGeoBIF website . To develop the TAPIR indexer we have relied on processes developed through the TapirTester software . All indexed data and georeferencing results are stored locally using PostgreSQL.

The user interactions and many other automated parts of the workflow occur entirely within a set of modules for the PHP-based Drupal web framework. There are several distinct benefits to this approach. First, it keeps our system highly modular. For example, for each type of data access point (DiGIR, TAPIR, BioCASE, etc.) we can deploy a self contained module within our website to handle both resource mapping and record harvesting. It also allows the administrator to completely change the look and layout of the service with minimal redevelopment cost. The modular approach will also make our service extremely versatile in the face of new technologies. Should a ubiquitous data indexer become available in the near future, we can divert development from harvesting to other aspects of the project by plugging the ubiquitous harvester into our workflow.

## Competing interests

The authors declare that they have no competing interests.

## Authors' contributions

This work was collaboratively conceived by RB, PF, AH and RPG and was further developed through conversations with VC and DR. AH, AR, PF, RB and RPG carried out the design of the project. AH has been developing the code and implementing the project in consultation with all co-authors. All authors contributed to the writing of the manuscript and have read and approved the final manuscript.
